# Targeting Glycolysis with Epigallocatechin-3-Gallate Enhances the Efficacy of Chemotherapeutics in Pancreatic Cancer Cells and Xenografts

**DOI:** 10.3390/cancers11101496

**Published:** 2019-10-05

**Authors:** Ran Wei, Robert M. Hackman, Yuefei Wang, Gerardo G. Mackenzie

**Affiliations:** 1 Tea Science Institute, Zhejiang University, Hangzhou 310058, China; wran@ucdavis.edu (R.W.); zdcy@zju.edu.cn (Y.W.); 2 Department of Nutrition, University of California, Davis, CA 95616, USA; rmhackman@ucdavis.edu; 3 Davis Comprehensive Cancer Center, University of California, Sacramento, CA 95817, USA

**Keywords:** pancreatic cancer, epigallocatechin-3-gallate (EGCG), gemcitabine, glycolysis, ROS, phosphofructokinase

## Abstract

Pancreatic cancer is a complex disease, in need of new therapeutic approaches. In this study, we explored the effect and mechanism of action of epigallocatechin-3-gallate (EGCG), a major polyphenol in green tea, alone and in combination with current chemotherapeutics on pancreatic cancer cell growth, focusing on glycolysis metabolism. Moreover, we investigated whether EGCG’s effect is dependent on its ability to induce reactive oxygen species (ROS). EGCG reduced pancreatic cancer cell growth in a concentration-dependent manner and the growth inhibition effect was further enhanced under glucose deprivation conditions. Mechanistically, EGCG induced ROS levels concentration-dependently. EGCG affected glycolysis by suppressing the extracellular acidification rate through the reduction of the activity and levels of the glycolytic enzymes phosphofructokinase and pyruvate kinase. Cotreatment with catalase abrogated EGCG’s effect on phosphofructokinase and pyruvate kinase. Furthermore, EGCG sensitized gemcitabine to inhibit pancreatic cancer cell growth in vitro and in vivo. EGCG and gemcitabine, given alone, reduced pancreatic tumor xenograft growth by 40% and 52%, respectively, whereas the EGCG/gemcitabine combination reduced tumor growth by 67%. EGCG enhanced gemcitabine’s effect on apoptosis, cell proliferation, cell cycle and further suppressed phosphofructokinase and pyruvate kinase levels. In conclusion, EGCG is a strong combination partner of gemcitabine reducing pancreatic cancer cell growth by suppressing glycolysis.

## 1. Introduction

Pancreatic cancer is one of the top five causes of cancer-related death in the United States, with an overall five-year survival rate of approximately eight percent [1]. While surgery, which offers the only realistic hope, is a viable option in a limited number of patients, current radiation therapy and chemotherapy regimens offer no significant clinical benefit [2]. For example, gemcitabine is an antimetabolite recognized as the current standard of care for unresectable locally advanced or metastatic pancreatic cancer, but its therapeutic effect for patients is limited [3]. Thus, an urgent need exists for new ways to combat pancreatic cancer, with the exploration of novel therapeutic strategies being a critical component.

Deregulated energy metabolism is recognized as one of the hallmarks of cancer [4]. Unlike normal cells, tumor cells prefer to rely on aerobic glycolysis to produce energy and to obtain intermediate metabolites that can then be directed to other biosynthetic pathways that promote tumor growth [5,6]. Moreover, the higher glycolytic rate in cancer cells has been shown to correlate to chemotherapeutic resistance [7]. Therefore, targeting tumor glycolysis remains attractive for therapeutic interventions, not only as a main target, but also for its ability to sensitize cancer cells to chemotherapeutics.

Epigallocatechin-3-gallate (EGCG), the most active polyphenol component found in green tea, presents several anticancer properties [8,9,10]. Accumulating evidence, including a previous study by our group, has shown that EGCG can inhibit cancer cell growth by modulating metabolic pathways [11,12,13]. An important consideration when exploring EGCG’s mechanism of action is that in the presence of oxygen, EGCG undergoes auto-oxidation to generate reactive oxygen species (ROS) [14]. Since cancer cells are more susceptible to an increase in ROS levels [15], this has been proposed as a potential mechanism of action for the inhibitory cell growth effect by EGCG. However, to date, whether the glycolytic pathway is modulated by EGCG in pancreatic cancer, and whether it is ROS-dependent is not completely elucidated.

In the present study, we explored the effect and mechanism of action of EGCG alone and in combination with current chemotherapeutics on pancreatic cancer cell growth, focusing on glycolysis metabolism involved in pancreatic cancer cell growth, and investigated whether it is dependent on the ability of EGCG to induce ROS. Our results show that EGCG strongly reduced pancreatic cancer cell growth by suppressing glycolysis in a ROS-dependent manner. Moreover, EGCG enhanced the anticancer effect of gemcitabine in vitro and in vivo by inhibiting glycolysis and affecting cell kinetics.

## 2. Results

### 2.1. EGCG Affects Glycolysis in Pancreatic Cancer Cells

**Figure 1:**
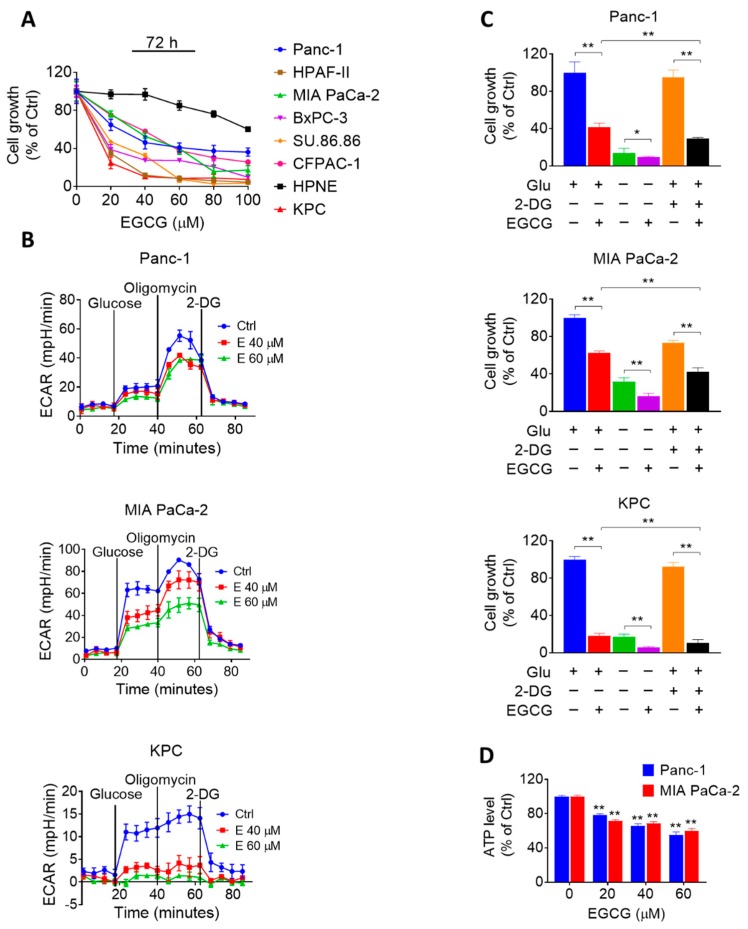
Epigallocatechin-3-gallate (EGCG) inhibits pancreatic cancer cell growth through glycolysis suppression. (**A**) EGCG inhibits human pancreatic cancer cell growth in a concentration-dependent manner. Cell growth was determined in Panc-1, MIA PaCa-2, HPAF-II, BxPC-3, SU-86.86, CFPAC-1, and KPC pancreatic cancer cells, and in the human pancreatic normal epithelial (HPNE) cells after treatment with increasing EGCG concentrations for 72 h. Results are expressed as a percentage of control. (**B**) EGCG suppresses glycolysis capacity in Panc-1, MIA PaCa-2, and KPC cells after 24 h. Glucose (25 mM), Oligomycin (1 µM) and 2-Deoxy-D-glucose (2-DG) (75 mM) were injected and the extracellular acidification rate (ECAR) of live cells was monitored during the experimental period. Results are presented as the mean ± SD of ECAR. (**C**) Cell growth was measured in Panc-1, MIA PaCa-2, and KPC cells treated with or without EGCG (40 µM) under glucose deprivation or 2-DG (10 mM) treatment condition. Results are expressed as a percentage of control. * *p* < 0.05, ** *p* < 0.01 vs. control. (**D**) EGCG reduced ATP levels in Panc-1 and MIA PaCa-2 cells after 24 h. Results are expressed as a percentage of control. * *p* < 0.05, ** *p* < 0.01 vs. control.

We initially evaluated the efficacy of EGCG on pancreatic cancer cells in culture. For this purpose, we treated multiple human pancreatic cancer cells with or without increasing concentrations of EGCG (20–100 μM) for 72 h. In parallel, we also compared the effect of EGCG in human pancreatic cancer cells to that of human pancreatic normal epithelial cells (HPNE). EGCG reduced human pancreatic cancer cell growth in a concentration-dependent manner. Of note, EGCG reduced cell growth more potently in human pancreatic cancer cells compared to HPNE cells. For instance, at 72 h, EGCG 80 µM reduced MIA PaCa-2 and Panc-1 cell growth by 84% and 64% (*p* < 0.05), respectively. In contrast, EGCG at 80 µM for 72 h had significantly less effect on the HPNE cells, reducing cell growth by only 24% (Figure 1A).

Given that alterations in cancer cell metabolism can lead to an inhibition of cell growth [16], and because EGCG has been shown to affect glycolysis in other tumor types [11,12,13], we evaluated the effect of EGCG on glucose metabolism in two human pancreatic cancer cells and one murine pancreatic cancer cell line (KPC). We assessed the impact of EGCG on glycolysis by measuring the extracellular acidification rate (ECAR) in the MIA PaCa-2, Panc-1, and KPC cell lines. After 24 h of treatment, EGCG strongly reduced ECAR in all three cell lines, revealing an inhibitory effect of EGCG on glycolysis (Figure 1B and Figure S1).

Next, we determined the effect of EGCG on pancreatic cancer cell growth under the conditions of either glucose deprivation or treatment with 2-Deoxy-D-glucose (2-DG), a glucose analog. As shown in Figure 1C, the reduction in cell growth induced by EGCG was enhanced under glucose deprivation or 2-DG treatment. For example, 2-DG alone reduced the cell growth rate by 5%, 27%, and 8% in Panc-1, MIA PaCa-2, and KPC cells, respectively, whereas when combined with EGCG, the cell growth was reduced by 71%, 58%, and 89%, respectively (*p* < 0.01; Figure 1C). Moreover, EGCG treatment reduced ATP levels concentration-dependently (Figure 1D). Treatment with EGCG at 40 μM for 24 h reduced ATP levels in Panc-1 and MIA PaCa-2 cells by 35% and 32%, respectively (*p* < 0.01 for both, Figure 1D).

### 2.2. EGCG Inhibits Glycolysis through Suppressing Rate-Limiting Enzymes

**Figure 2:**
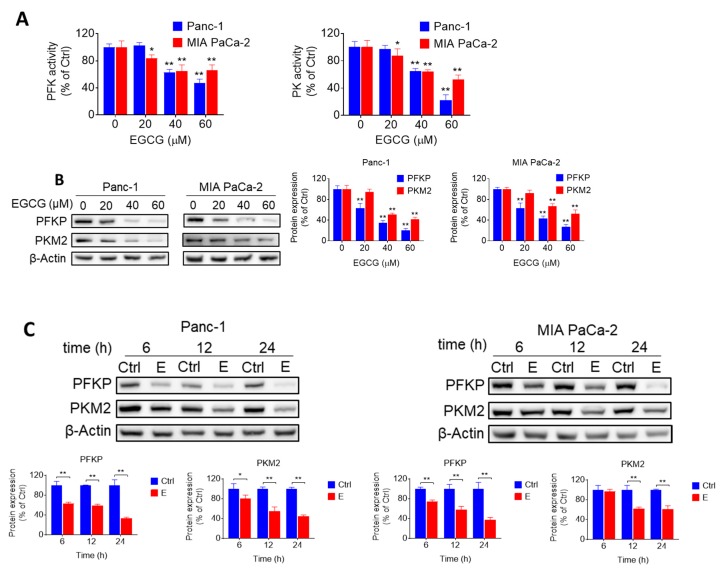

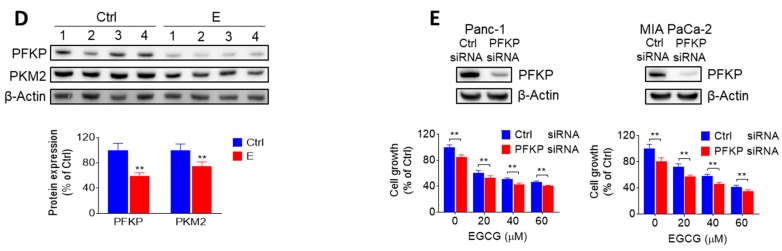
EGCG inhibits glycolysis through suppressing rate-limiting enzyme activity and expression. (**A**) Phosphofructokinase (PFK) and pyruvate kinase (PK) activities were determined in Panc-1 and MIA PaCa-2 cells after treatment with EGCG for 24 h. Results are expressed as a percentage of control. * *p* < 0.05, ** *p* < 0.01 vs. control. (**B**) Immunoblots for platelet-type phosphofructokinase (PFKP) and pyruvate kinase M2 (PKM2) in total cell protein extracts from Panc-1 and MIA PaCa-2 cells treated with escalating concentrations of EGCG, as indicated, for 24 h. Loading control: β-Actin. Bands were quantified and results are expressed as a percentage of control. * *p* < 0.05, ** *p* < 0.01 vs. control. (**C**) EGCG (40 µM) inhibited PFKP and PKM2 protein expression in a time-dependent manner in Panc-1 and MIA PaCa-2 cells. Results are expressed as percentage of control and presented as the mean ± SD. * *p* < 0.05, ** *p* <0.01 vs. control. (**D**) Immunoblots of PFKP, PKM2 expression on tumor tissue from control- and EGCG-treated (10mg/kg/d) mice. Results are expressed as a percentage of control. * *p* < 0.05, ** *p* < 0.01 vs. control. (**E**) Effect of silencing PFKP on EGCG-induced cell growth reduction. Panc-1 and MIA PaCa-2 cells were transfected with either control or PFKP siRNA. After transfection, cells were treated with EGCG for 72 h and cell growth was evaluated. Results are expressed as percentage of control; * *p* < 0.05, ** *p* < 0.01 vs. control. Immunoblots to verify PFKP silencing were performed on whole cell extracts obtained from these cells (top panel).

Given the effect of EGCG on glycolysis, we evaluated whether EGCG could affect any particular step in the glycolytic pathway by measuring the activity and levels of glycolytic enzymes. EGCG treatment reduced both the activity and expression levels of phosphofructokinase (PFK) and pyruvate kinase (PK) in Panc-1 and MIA PaCa-2 cells, having a stronger effect on PFK (Figure 2A–C, Figure S5). For instance, EGCG at 40 μM reduced the levels of platelet-type phosphofructokinase (PFKP) and the pyruvate kinase M2 (PKM2), an isoform of PK, by 65% and 49%, respectively, in Panc-1 cells, and by 57% and 34%, respectively in MIA PaCa-2 cells (Figure 2B, Figure S4). However, EGCG failed to reduce hexokinases II (HK2) and lactate dehydrogenase A (LDHA) protein expression levels (Figure S2, Figure S12). In agreement with the in vitro results, EGCG reduced the levels of PFKP and PKM2 (*p* < 0.01 for both) in pancreatic tumor xenograft homogenates, obtained from mice treated with EGCG (Figure 2D, Figure S6).

To confirm the role of PFKP on EGCG-induced cell growth inhibition, we silenced PFKP in Panc-1 and MIA PaCa-2 cells. Knocking-down PFKP reduced Panc-1 and MIA PaCa-2 cell growth by 15% and 19%, respectively. In Panc-1 and MIA PaCa-2 cells transfected with nonspecific siRNA, EGCG 40 μM decreased the number of viable cells by 49% and 42%, whereas in PFKP-silenced cells, EGCG reduced cell growth by 57% and 54% in Panc-1 and MIA PaCa-2 cells, respectively (Figure 2E, Figure S6). Taken together, these findings indicate that silencing PFKP function has a slight additive effect on the growth inhibitory response of EGCG in both cell lines, and suggest that regulating glycolysis represents an important mechanism of EGCG in inhibiting pancreatic cancer cell growth.

### 2.3. EGCG Affects the Glycolytic Pathway in Pancreatic Cancer Cells through a ROS-Dependent Manner

**Figure 3:**
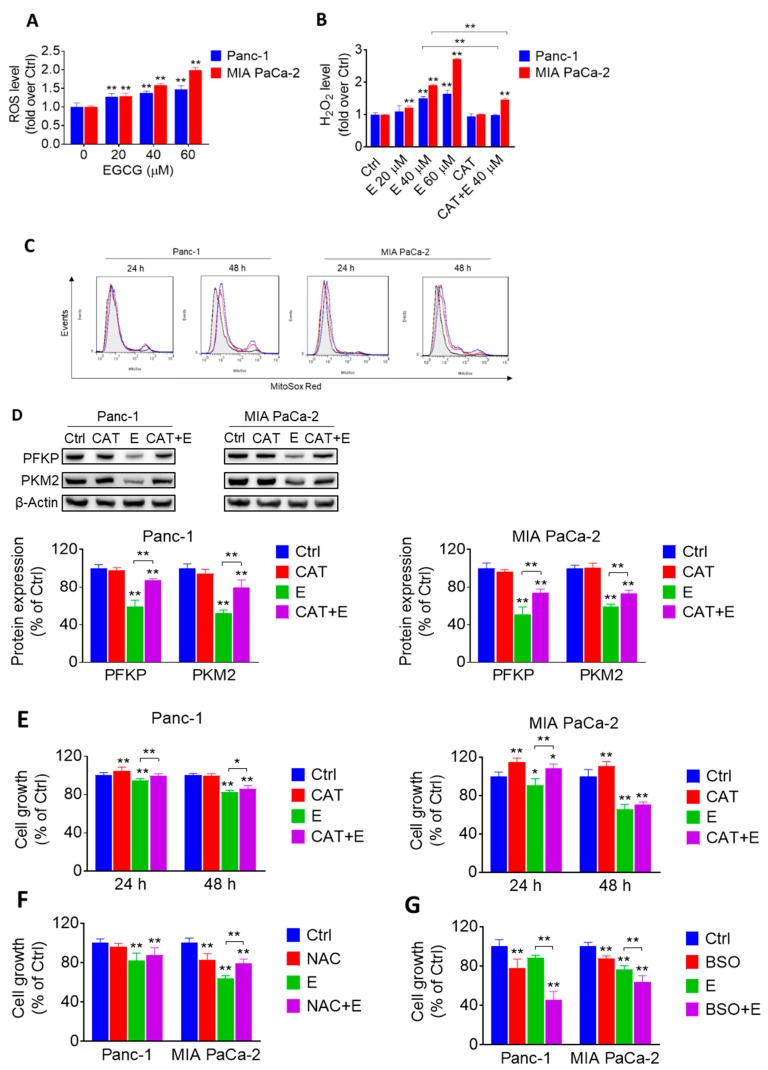
EGCG affects the glycolytic pathway in pancreatic cancer through a reactive oxygen species (ROS)-dependent manner. (**A**) 2′,7′-Dichlorodihydrofluorescein diacetate (H2DCFDA) fluorescence was measured in Panc-1 and MIA PaCa-2 cells treated without (control) or with EGCG at various concentration for 24 h. Results are expressed as fold change of control. * *p* < 0.05, ** *p* < 0.01 vs. control. (**B**) Amplex^TM^ Red was used to evaluate the levels of hydrogen peroxide level released by Panc-1 and MIA PaCa-2 cells. Results are expressed as fold change of control. * *p* < 0.05, ** *p* < 0.01 vs. control. (**C**) EGCG induces a concentration- and time-dependent increase in mitochondrial superoxide anion levels. The levels of superoxide anion in the mitochondria were determined by flow cytometry using the MitoSOX-Red fluorescent probe. Black, red and blue lines represent control, EGCG 40 μM, and EGCG 60 μM groups, respectively. (**D**) The reduction in the expression levels of PFKP and PKM2 triggered by EGCG (E) was reversed by catalase (CAT). Immunoblots for PFKP and PKM2 in total cell protein extracts from Panc-1 and MIA PaCa-2 cells treated with 40 µM EGCG (E), 1500 U/mL catalase (CAT) or both, for 12 h. Loading control: β-Actin. Bands were quantified and results are expressed as a percentage of control. * *p* < 0.05, ** *p* < 0.01 vs. control. (**E**) Catalase (CAT) partly ameliorated the cell growth inhibitory effect induced by EGCG (E). Cells were treated with 40 µM EGCG (E), 1500 U/mL CAT or both, for 24 h or 48 h. Results are expressed as a percentage of control. * *p* < 0.05, ** *p* < 0.01 vs. control. (**F**) N-Acetyl-L-Cysteine (NAC) partly reversed cell inhibition effect of EGCG after 48 h. Cells were treated with 40 µM EGCG (E), 5 mM NAC or both for 48 h. Results are expressed as percentage of control. * *p* < 0.05, ** *p* < 0.01 vs. control. (**G**) L-Buthionine Sulfoximine (BSO) further aggravated cell inhibition effect of EGCG after 24 h. Cells were treated with 40 µM EGCG (E), 1 mM BSO or both for 24 h. Results are expressed as a percentage of control. * *p* < 0.05, ** *p* < 0.01 vs. control.

Because EGCG and other polyphenols have been shown to undergo rapid oxidization to generate free radicals in the presence of oxygen [17], we evaluated the ability of EGCG to induce reactive oxygen species (ROS) in pancreatic cancer cells. Compared to the control, treatment with EGCG significantly increased ROS levels concentration-dependently, as determined with the general probe 2′,7′-Dichlorodihydrofluorescein diacetate (H2DCFDA). For example, EGCG at 40 µM increased ROS levels by 1.4- and 1.6-fold in Panc-1 and MIA PaCa-2 cells, respectively (*p <* 0.01 for both; Figure 3A).

Next, we determined the levels of hydrogen peroxide (H_2_O_2_) using the Amplex™ Red indicator probe. EGCG strongly increased H_2_O_2_ levels in pancreatic cancer cells. Compared to controls, EGCG at 40 µM increased H_2_O_2_ levels by 1.5- and 1.9-fold in Panc-1 and MIA PaCa-2 cells, respectively (Figure 3B). In both cell lines, cotreatment with catalase significantly abrogated the increase in H_2_O_2_ levels induced by EGCG (Figure 3B). Moreover, EGCG increased the levels of mitochondria superoxide anion in a concentration and time-dependent manner in both Panc-1 and MIA PaCa-2 cells (Figure 3C).

To elucidate whether the effect of EGCG on glycolysis is mediated through the increase in ROS, Panc-1 and MIA PaCa-2 cells were incubated with or without EGCG at 40 µM in the presence or absence of catalase for 12 h. In both cell lines, catalase mostly abrogated the inhibitory effect of EGCG on PFKP and PKM2, suggesting that the effect of EGCG on glycolytic enzymes is dependent on the ability of EGCG to induce ROS (Figure 3D, Figure S7).

However, catalase could only, in part, prevent the reduction in pancreatic cancer cell growth induced by EGCG (Figure 3E). Somewhat similar results were obtained when cotreating with N-Acetyl-L-Cysteine (NAC) (Figure 3F). In contrast, treatment with L-Buthionine Sulfoximine (BSO), an inhibitor of glutamylcysteine synthetase, strongly enhanced the reduction in cell growth induced by EGCG in both cell lines (Figure 3G). For example, in Panc-1 cells at 24 h, BSO and EGCG alone reduced cell growth by 22% and 12%, respectively, and by 54% when combined. These results suggest that EGCG reduces pancreatic cancer growth, partly, through an ROS-dependent mechanism.

### 2.4. EGCG Sensitizes Pancreatic Cancer Cells to Gemcitabine In Vitro

**Figure 4:**
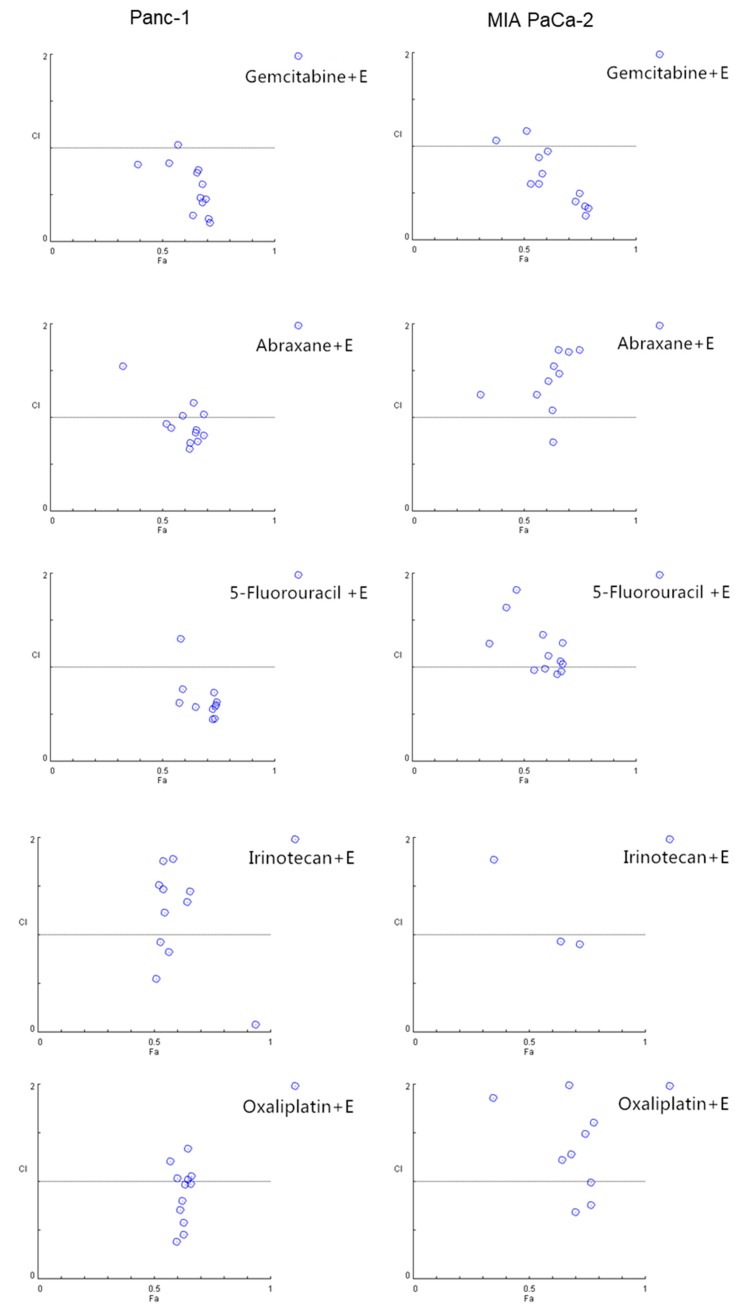
EGCG enhances the cell growth inhibitory effect of gemcitabine in pancreatic cancer cells. Combination index (CI) plots of EGCG (20, 40, 60 µM) in combination with gemcitabine (1, 10, 20 and 40 nM), abraxane (1, 10, 20 and 40 nM), 5-Fluorouracil (1, 10, 20 and 40 µM), irrinotecan (1, 10, 20 and 40 µM), and oxialiplatin (1, 10, 20 and 40 µM) in Panc-1 (left) and MIA PaCa-2 (right) cells. Drug interactions were quantitatively determined using the Chou–Talalay method, and CI < 1, =1, and >1 indicates synergism, additive and antagonism effect, respectively. Of note, the CI value dots depicted are based on concentrations of EGCG and the chemotherapeutic drugs shown on Table S1.

Because chemotherapeutics often display limited effects, show resistance, and cause side-effects, we evaluated whether EGCG would represent a useful partner in combination with commonly used drugs. For this purpose, we treated Panc-1 and MIA PaCa-2 cells with EGCG alone or in combination with gemcitabine, Abraxane^®^, 5-Fluorouracil, irinotecan, or oxaliplatin, five commonly used chemotherapeutics in pancreatic cancer patients, for 72 h and analyzed their combination index (CI) by means of the Chou–Talalay method. In both Panc-1 and MIA PaCa-2 cells, EGCG strongly synergized with gemcitabine (Figure 4 and Table S1). For example, in Panc-1 cells, the CI of all tested groups, except one, showed synergistic effects. In MIA PaCa-2 cells, the CI effects between EGCG and gemcitabine were also indicative of a strong additive effect (Figure 4). The combination effect of EGCG with the other four chemotherapeutics tested presented effects that were more variable with some showing an additive effect (Figure 4 and Table S1).

### 2.5. EGCG Enhances the Anticancer Effect of Gemcitabine in Pancreatic Cancer Xenografts through a Strong Cytokinetic Effect

**Figure 5:**
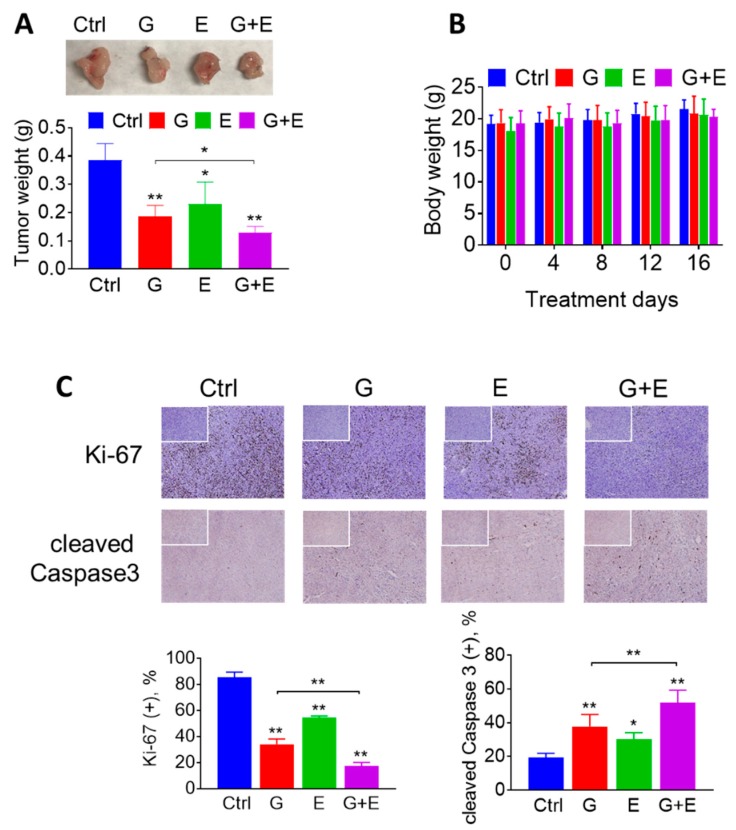
EGCG enhances the anticancer effect of gemcitabine in pancreatic cancer xenografts. (**A**) EGCG (E) promoted tumor weight lost with gemcitabine (G). Results are presented as the mean ± SD. * *p* < 0.05, ** *p* < 0.01 vs. control. (**B**) Mice body weight during treatment days for control, gemcitabine-treated groups (G), EGCG-treated groups (E), and the combination (G + E). Results are presented as the mean ± SD. (**C**) Immunostaining of the cell proliferation marker Ki-67 and of cleaved Caspase 3 were performed on KPC tumor sections and photographs were taken at 100× magnification. Representative images are shown. The consecutive section was stained with isotype Immunoglobulin G (IgG) as negative staining control and it is shown in the upper left corner. Results were expressed as percent of positive (+) cells ± SD per 100x field. * *p* < 0.05, ** *p* < 0.01 vs. control.

Next, we evaluated whether EGCG could enhance the chemotherapeutic effect of gemcitabine in vivo. For this purpose, murine pancreatic cancer KPC cells were injected subcutaneously into immunocompetent mice, which gave rise to exponentially growing tumors. Once the tumors reached ~300 mm^3^, the mice were treated either with EGCG (10 mg/kg) suspended in phosphate buffered saline (PBS) pH 7.4 given intraperitoneally (I.P.) once daily, gemcitabine (100 mg/kg) given I.P. twice per week, or both drugs. On day 16 of treatment, EGCG and gemcitabine, given as single agents, reduced tumor weight by 40% and 52%, respectively, compared to vehicle-treated controls (*p* < 0.05 and *p* < 0.01). In combination, EGCG plus gemcitabine reduced tumor weight by 67%, compared to controls (*p* < 0.01). Of note, the effect of the EGCG plus gemcitabine combination was significantly different from that of gemcitabine alone (*p* < 0.05; Figure 5A). Importantly, the drug combination was well tolerated by the mice, as indicated by the comparable mean body weight of the experimental groups throughout the treatment period (Figure 5B).

Because EGCG has been shown to be hepatotoxic at higher doses [18], and to further evaluate the safety of EGCG plus gemcitabine, we performed an acute toxicity study and determined the levels of multiple liver enzymes and kidney markers. After 16 days of EGCG plus gemcitabine treatment, liver and kidney function markers (including activity of Alanine Transaminase, Alkaline Phosphatase, Aspartate Transaminase, and the levels of blood urea nitrogen, creatinine, etc.) were in the normal range (Table S2). Consistent with the efficacy study, the mean body weights were comparable between the groups (Figure S3).

To investigate the mechanism by which EGCG plus gemcitabine reduced tumor growth, we determined cell proliferation (Ki-67 expression), and apoptosis (cleaved Caspase 3 expression) levels by immunohistochemistry in tumor tissue sections from control and EGCG plus/minus gemcitabine-treated mice (Figure 5C). Compared to controls, EGCG alone and EGCG plus gemcitabine inhibited cell proliferation (Ki-67 positive cells) by 45% and 83% in the KPC xenografts, respectively (*p* < 0.01, Figure 5C). In addition, EGCG increased the percentage of cleaved Caspase 3 positive cells by 1.6-fold when given alone and by 2.7-fold when given in combination (*p* < 0.01, Figure 5C).

**Figure 6:**
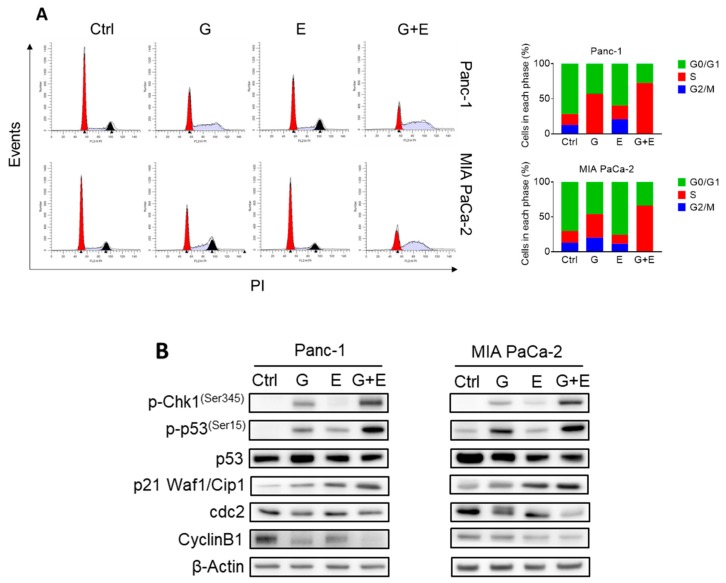

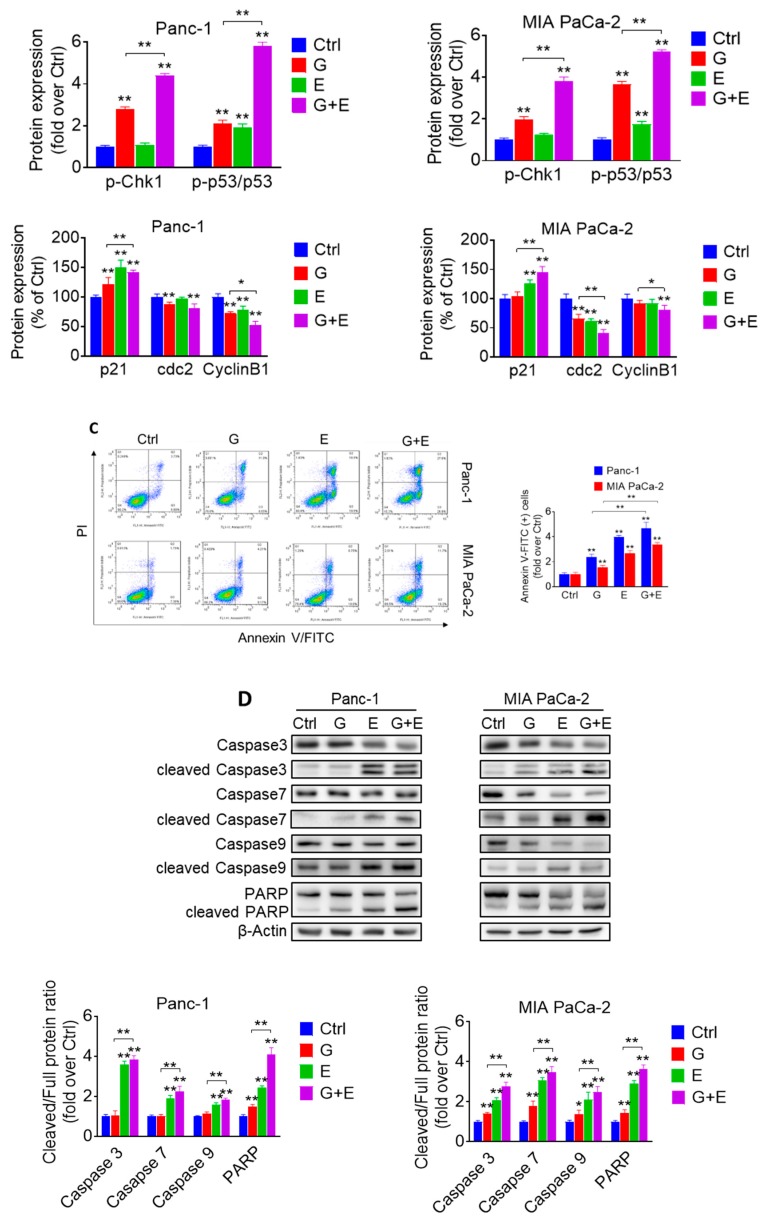

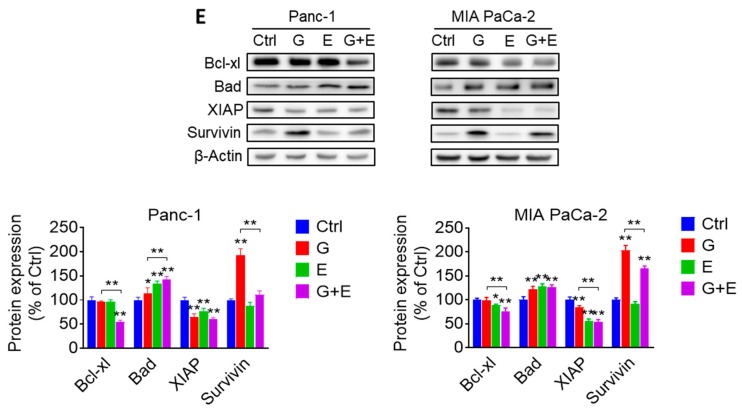
EGCG and gemcitabine together further affected cell kinetic in pancreatic cancer cells. (**A**) EGCG (E) enhanced the effect of gemcitabine (G) on the cell cycle. Following treatment with 40 µM EGCG (E), 20 nM gemcitabine (G), or both (G + E) for 48 h, cells were stained with propidium iodide (PI) and the number of cells in each phase of the cell cycle was measured by flow cytometry. Results are expressed as a percentage of control. (**B**) EGCG (E) and gemcitabine (G) modulated S/G2 phase protein expression. Immunoblots for phosphorylated checkpoint kinases 1 (p-Chk1), phosphorylated and total tumor protein p53 (p53), cyclin-dependent kinase (cdk) inhibitor p21 Waf1/Cip1 (p21), cell division cycle 2 (cdc2) and Cyclin B1 in total cell protein extracts from Panc-1 and MIA PaCa-2 cells treated with EGCG (E), gemcitabine (G), or both (G + E), for 48 h. Loading control: β-Actin. Bands were quantified and results are expressed as a percentage of control. * *p* < 0.05, ** *p* < 0.01 vs. control. (**C**) Panc-1 and MIA PaCa-2 cells were treated with EGCG (E), gemcitabine (G), or both (G + E) for 48 h, and the percentage of apoptotic cells were determined by flow cytometry using dual staining (Annexin V and propipium iodide). The percentages of Annexin V (+) cells was calculated, and results are expressed as the fold-increase over control. Co-treatment with EGCG (E) further increased the apoptosis rate induced by gemcitabine (G) alone after 48 h. Results are expressed as percentage of control. * *p* < 0.05, ** *p* < 0.01 vs. control. (**D**) Immunoblots for full length and cleaved Caspases 3, 7 and 9 as well as full length and cleaved poly (ADP-ribose) polymerase (PARP) in total cell protein extracts from Panc-1 and MIA PaCa-2 cells treated with EGCG (E), gemcitabine (G), or both (G + E) for 48 h. Loading control: β-Actin. Bands were quantified and results are shown as the ratio between the cleaved/full length protein; * *p* < 0.05, ** *p* < 0.01 vs. control. (**E**) EGCG sensitized gemcitabine on apoptosis induction by regulating B-cell lymphoma 2 (Bcl-2) family and Inhibitors of apoptosis proteins (IAP) family protein expression in Panc-1 and MIA PaCa-2 cells after 48 h. Results are expressed as percentage of control. * *p* < 0.05, ** *p* < 0.01 vs. control.

In vitro, we determined the effect of EGCG and gemcitabine on cell cycle progression and cell death by apoptosis. As expected, gemcitabine, an inhibitor of DNA synthesis [19], strongly induced Synthesis/Gap2 (S/G2) phase arrest after 48 h of treatment. While EGCG at 40 µM only slightly arrested cell cycle progression, it strongly enhanced the effect of gemcitabine, with the combination further blocking the cell cycle at S/G2 in Panc-1 and MIA PaCa-2 cells (Figure 6A). Concomitant with cell cycle arrest, expression levels of S/G2 checkpoint proteins, including phosphorylated checkpoint kinases 1 (p-Chk1), phosphorylated tumor protein p53 (p-p53), and cyclin-dependent kinase (cdk) inhibitor p21 Waf1/Cip1 (p21) further increased, while cdc2 and CyclinB1 were further reduced in the EGCG plus gemcitabine-treated cells compared to those treated with gemcitabine alone (Figure 6B, Figure S8).

Besides blocking cell cycle progression and consistent with the in vivo results, gemcitabine also induced apoptosis in pancreatic cancer cells in culture. Compared to controls, treatment with gemcitabine for 48 h resulted in a 2.4- and 1.6-fold increase in apoptosis in Panc-1 and MIA PaCa-2 cells, respectively (Figure 6C). While EGCG at 40 µM induced apoptosis by 3.9- and 2.7-fold, the effect of EGCG plus gemcitabine was enhanced 4.7- and 3.4-fold over control in Panc-1 and MIA PaCa-2 cells, a response that was approximately two times that of gemcitabine alone (*p* < 0.01).

We then determined the expression of proteins involved in the mechanism of apoptosis by Western blot. Compared to controls, EGCG plus gemcitabine significantly affected the expression of cleaved Caspase 3, 7, 9, cleaved poly (ADP-ribose) polymerase (PARP), proapoptotic member of the Bcl-2 family protein Bad, antiapoptotic member of the Bcl-2 family protein Bcl-xl, and X-linked inhibitor of apoptosis protein (XIAP) levels, but not survivin (Figure 6D,E and Figure S9). Of note, no additive effect was observed on the expression of cleaved Caspase 3, 7, 9, Bad, Bcl-xl, or XIAP between EGCG plus gemcitabine and EGCG alone (Figure 6E, Figure S10), suggesting that the effect of the combination is most likely being driven by EGCG.

### 2.6. EGCG Plus Gemcitabine Further Inhibits Glycolysis

**Figure 7:**
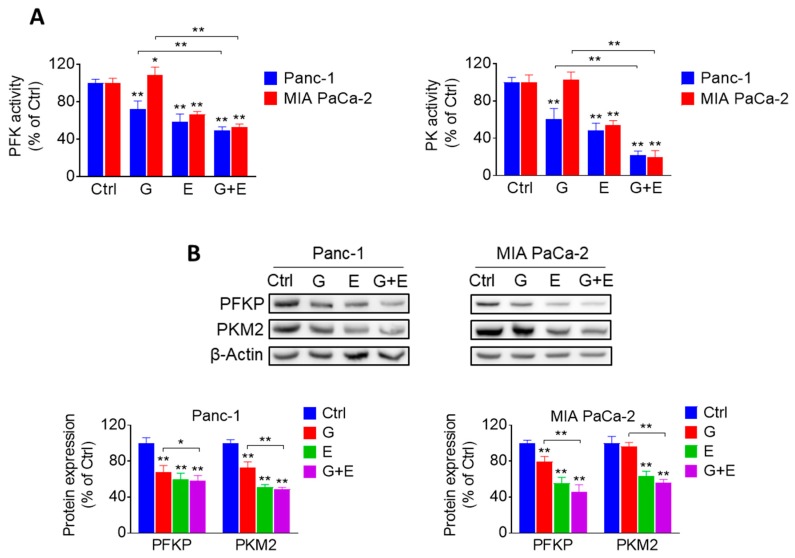
EGCG plus gemcitabine inhibit glycolysis. (**A**) The activity of PFK and PK was determined in Panc-1 and MIA PaCa-2 cells treated with 40 µM EGCG (E), 20 nM gemcitabine (G), or both (G + E) for 24 h. Results are expressed as a percentage of control. * *p* < 0.05, ** *p* < 0.01 vs. control. (**B**) Immunoblots for PFKP and PKM2 in total cell protein extracts from Panc-1 and MIA PaCa-2 cells treated with EGCG (E), gemcitabine (G), or both (G + E) for 12 h. Loading control: β-Actin. Bands were quantified and results are expressed as a percentage of control. * *p* < 0.05, ** *p* < 0.01 vs. control.

Given that EGCG strongly affected the glycolytic pathway, we evaluated whether combining EGCG with gemcitabine would lead to any additional glycolysis inhibitory effect. For this purpose, we determined the activity and levels of glycolytic enzymes in Panc-1 and MIA PaCa-2 cells, treated with EGCG and gemcitabine alone or in combination. The activity and levels of PFK and PK were significantly reduced compared to controls and gemcitabine alone groups. However, the effect of the combination group was not significant compared to the EGCG group (Figure 7A,B and Figure S11).

## 3. Discussion

Pancreatic cancer continues to be a significant health problem around the world. Given the lack of effective treatments that can meaningfully prolong a patient’s life, an active search for agents that have additive or synergistic effect with chemotherapeutic drugs is critical in order to enhance their efficacy. In this study, we show that EGCG sensitizes pancreatic cancer cells to gemcitabine by suppressing glycolysis.

Our work identified the glycolytic pathway as one of the major signaling mechanisms involved in eliciting the growth inhibitory effect of EGCG. Glycolysis supports cell growth by rapidly generating ATP and metabolic intermediates for other biosynthetic pathways. A high rate of glycolysis is characteristic of cancer cells, even in the presence of sufficient oxygen [20]. EGCG strongly inhibited the glycolytic rate in pancreatic cancer cells by affecting the activity and expression of PFK and PK, two essential rate-limiting enzymes involved in glycolysis. The finding that glucose deprivation or 2-DG treatment enhances the growth inhibition effect of EGCG indicates that glycolysis is related to pancreatic cancer cell growth.

PFK catalyzes the first irreversible reaction of glycolysis and is usually highly expressed in tumor tissues. The platelet isoform of PFK, PFKP, functions as an important mediator in cancer cell proliferation and metastasis [21,22]. On the other hand, PKM2, an isoform of PK, is involved in regulating cell growth and metastasis [23]. Our results showed that EGCG decreased the enzyme activity and protein expression of PFK and PK in vitro and in vivo, but not HK2 or LDHA. Of note, silencing PFKP had a slight additive effect on the growth inhibitory effect of EGCG, suggesting that regulating glycolysis represents an important mechanism of EGCG in inhibiting pancreatic cancer cell growth. These results are consistent with other studies that also report inhibition of growth through the suppression of glycolysis by EGCG, such as in breast and hepatocellular cancer cell models [11,12].

The induction of oxidative stress plays a significant role in the effect of many anticancer agents [24]. Compared with normal cells, cancer cells exhibit higher levels of ROS and antioxidant levels to maintain redox homeostasis. Cancer cells are thus more susceptible to oxidative stress [15], which precedes the induction of apoptotic cell death [25]. EGCG induced ROS levels in pancreatic cancer cells in culture, consistent with its known pro-oxidant activity [26]. Interestingly, the inhibitory effect of EGCG on PFKP and PKM2 levels was mostly reversed by catalase, suggesting that the effect of EGCG on glycolytic processes is ROS-dependent. However, catalase only partly prevented the growth inhibition effect of EGCG, indicating that the ROS produced by EGCG could only explain part of the growth inhibitory effect by EGCG. Interestingly, we have recently shown that EGCG inhibits the protein kinase B (PKB, also known as Akt) pathway though a ROS-independent effect [27]. Thus, the effect of EGCG on the growth of pancreatic cancer cells appears to be the result of the sum of EGCG’s ROS-dependent and independent effects. Moreover, the significance of oxidative stress in the reduction of cell growth can be further evidenced by two manipulations of the system affecting the levels of glutathione. Pretreatment with BSO, which depletes intracellular glutathione, strongly enhanced the growth inhibitory effect of EGCG. Alternatively, supplementing the cells with NAC attenuated the growth inhibitory effect of EGCG.

A common practice in the clinic is to administer multiple drugs concomitantly to cancer patients to help enhance a beneficial effect at lower doses while reducing side effects. The combination of gemcitabine with Abraxane^®^ is the most widely used regimen for patients with newly diagnosed pancreatic cancer [28]. Another option is the combined therapy of leucovorin-modulated 5-Fluorouracil (5-FU), irinotecan, and oxaliplatin (FOLFIRINOX). Unfortunately, this regimen is given only to patients that can tolerate its toxicity, which includes a higher degree of neutropenia, diarrhea, and sensory neuropathy [29]. In this work, we explored whether EGCG could enhance the anticancer efficacy of the Food and Drug Administration (FDA)-approved drugs mentioned above. EGCG successfully enhanced the cell growth inhibition effect of gemcitabine in vitro and in vivo. Importantly, EGCG plus gemcitabine, at their effective doses, appeared to be safe to mice, being well tolerated and showing no signs of liver toxicity. As an analog of deoxycytidine, gemcitabine enters the cells, embeds into DNA, inhibits DNA synthesis, and induces cell cycle arrest. EGCG enhanced gemcitabine’s suppression of cell growth through the inhibition of cell proliferation, the arrest of the cell cycle, and the induction of apoptosis through activation of execution Caspases [30], modulation of Bcl-2, and inhibition of apoptosis protein families. Mechanistically, the two agents together showed an additive effect of inhibiting glycolysis in pancreatic cancer cells. Of note, additional studies in complementary preclinical pancreatic cancer models are needed to validate the anti-tumor effect of EGCG with gemcitabine and advance the preclinical development of this drug combination. In summary, these results suggest the possibility to utilize EGCG as a useful adjuvant drug with gemcitabine to inhibit pancreatic cancer by further modulating cell kinetics and suppressing glycolysis.

## 4. Materials and Methods 

### 4.1. Chemicals and Reagents

EGCG (≥98% purity) was purchased from Tocris (Minneapolis, MN, USA). 3-(4,5-dimethylthiazol-2-yl)-2,5-diphenyltetrazolium bromide (MTT) (≥97.5%), D-(+)-Glucose (≥99.5%), Oligomycin (≥90%), 2-Deoxy-d-glucose (≥99%), Irinotecan hydrochloride, Oxaliplatin, Catalase, *N*-Acetyl-l-cysteine (≥99%), DL-Buthionine-sulfoximine (≥99%), RIPA lysis buffer, Halt Protease Inhibitor Cocktail, Phosphatase Inhibitor Cocktail, and the following kits: Phosphofructokinase Activity Colorimetric assay and Pyruvate Kinase Activity assay were purchased from MilliporeSigma (St. Louis, MO, USA). Seahorse XF24 Extracellular Flux assay kits were purchased from Agilent (Santa Clara, CA, USA). CellTiter-Glo^®^ reagent and rATP were purchased from Promega (Madison, WI, USA). PFKP siRNA plasmid was purchased from Santa Cruz Biotechnology (Dallas, TX, USA). Gemcitabine-HCL (>99%) was purchased from BIOTANG (Waltham, MA, USA). 5-Fluorouracil (≥99%) was purchased from Alfa Aesar (Haverhill, MA, USA). Annexin V-FITC conjugate, propidium iodide (PI), 2’,7’-dichlorodihydrofluorescein diacetate (H2DCFDA), Amplex^®^ Red Hydrogen Peroxide kit, MitoSOX™ Red Mitochondrial Superoxide Indicator, Lipofectamine™ 3000 Transfection reagent, and SuperSignal™ West Dura Extended Duration Substrate were purchased from ThermoFisher Scientific (Waltham, MA, USA). Antibodies for Western blot were purchased from Cell Signaling Technology (Danvers, MA, USA). Bradford protein assay reagent, 30% (*w*/*v*) Acrylamide/Bis Solution, 4xLaemmli sample buffer, and Immun-Blot^®^ PVDF Membranes were purchased from Bio-Rad (Hercules, CA, USA).

### 4.2. Cell Culture

Human pancreatic cancer cell lines (BxPC-3, HPAF-II, CFPAC-1, Su.86.86, Panc-1 and MIA PaCa-2), and human pancreatic normal epithelial (HPNE) cells were sourced from the American Type Culture Collection (Manassas, VA, USA). FC1245 cells (KPC, murine pancreatic cancer cells) were a gift from Dr. David Tuveson (Cold Spring Harbor Laboratory, Cold Spring Harbor, NY, USA). All cell lines were grown as monolayers in a specific medium under conditions suggested by the vendor. Although these cells lines were not authenticated in our lab, they were characterized by cell morphology and growth rate, and cultured in our laboratory less than six months after being received. We also routinely test for mycoplasma contamination in all cell lines every three months.

### 4.3. Cell Viability

Following the treatment with EGCG or the various chemotherapeutic drugs for 72 h, the reduction of 3-(4,5-dimethylthiazol-2-yl)-2,5-diphenyltetrazolium bromide dye (MTT) was determined according to the manufacture’s protocol (MilliporeSigma, St. Louis, MO, USA).

### 4.4. Cellular Glycolytic Rate Measurements

The cellular glycolytic rate, represented as the extracellular acidification rate (ECAR), was measured using a Seahorse XF24 Extracellular Flux Analyzer (Agilent, Santa Clara, CA, USA). Briefly, Panc-1 and MIA PaCa-2 cells, plated on XF24 cell culture plates, were incubated with the agents for 24 h, and then assayed with a glycolytic stress test kit, following the manufacturer’s instruction (Agilent, Santa Clara, CA, USA).

### 4.5. Cellular ATP Levels

Cells, plated in white 96-well plates, were treated with test drugs for 24 h. After treatment, ATP levels were measured with the CellTiter-Glo^®^ reagent, following the manufacturer’s protocol (Promega, Madison, WI, USA). A standard curve was generated using escalating concentrations of ATP.

### 4.6. Glycolysis-Related Enzymes Activity

Cells were seeded into the 6-well plates overnight and treated with EGCG for 24 h. Following the incubation, cells were collected, washed with cold PBS, and homogenized. After centrifugation, the supernatant was collected and used to measure the phosphofructokinase (PFK) and pyruvate kinase (PK) enzyme activities following the manufacturer’s protocol (MilliporeSigma, Saint Louis, MO, USA). Protein concentration was determined using the Bradford protein assay.

### 4.7. Gene Silencing

Cells were plated in 6-well plates overnight, and transiently transfected with PFKP siRNA or nonspecific control siRNA for several hours using Lipofectamine^TM^ 3000 reagent according to the manufacturer’s instructions (ThermoFisher Scientific, Waltham, MA, USA). Following transfection, cells were replated and treated with EGCG for up to 72 h and cell viability was tested. The gene silencing efficiency was determined by immunoblotting.

### 4.8. Determination of ROS Levels

After treatment with EGCG for 24 h, cells were incubated with 10 μM H2DCFDA for 30 min at 37 °C and their fluorescence intensity was analyzed using a Synergy H1 microplate reader (Biotek, Winooski, VT, USA). Hydrogen peroxide (H_2_O_2_) levels were detected using an Amplex™ Red hydrogen peroxide kit according to the manufacturer’s instruction (ThermoFisher Scientific, Waltham, MA, USA).

### 4.9. Mitochondrial Superoxide Level Analysis

After treatment with EGCG for 24 or 48 h, cells were collected and incubated with 5 μM MitoSOX^TM^ Red mitochondrial superoxide probe at 37 °C for 30 min. The fluorescence intensity was determined by FACScan flow cytometry (Becton Dickinson, San Jose, CA, USA) and results were analyzed with FlowJo software (v7.6, Tree Star, Inc., Ashland, OR, USA).

### 4.10. Cell Apoptosis

After cells were treated with the test agents in 6-well plates, they were trypsinized and stained with Annexin V-FITC (100× dilution) and PI (0.5 µg/mL) for 15 min. Annexin V-FITC and PI fluorescence intensities were analyzed by FACScan (Becton Dickinson, San Jose, CA, USA). Annexin V (+)/PI (-) cells are apoptotic cells, Annexin V (+)/ PI (+) cells have undergone secondary necrosis, and Annexin V (-)/ PI (+) cells are necrotic cells. Results were analyzed by using FlowJo software.

### 4.11. Cell Cycle Analysis

Cells were seeded in 6-well plates and treated for 48 h. After each treatment, cells were trypsinized and fixed in 70% (*v*/*v*) ethanol overnight, stained with PI (50 µg/mL) and RNase A (10 mg/mL) for 15 min, and subjected to flow cytometric analysis (Becton Dickinson, San Jose, CA, USA).

### 4.12. Western Blot

Whole cell protein lysates were prepared, and electrophoresis and electroblotting were performed as previously described [31]. Membranes were probed overnight with the following primary antibodies (1:1000 dilution) from Cell Signaling Technology (Danvers, MA, USA): PFKP (Cat #8164), PKM2 (Cat #4053), HK2 (Cat #2867), LDHA (Cat #3582), phospho-Chk1 (Ser345) (Cat #2348), phospho-p53 (Ser15) (Cat #9286), p53 (Cat #2527), p21 Waf1/Cip1 (Cat #2947), cdc2 (Cat #28439), Cyclin B1 (Cat #12231), Caspase-3 (Cat #14220), Caspase-7 (Cat #12827), Caspase-9 (Cat #9508), and PARP (Cat #9542). β-Actin (Cat #8457) was used at the same time as a loading control. After incubation for 60 min at room temperature in the presence of the secondary antibody (HRP-conjugated; 1:5000 dilution), the conjugates were developed and visualized using a Molecular Imager FX^TM^ System (BioRad; Hercules, CA, USA).

### 4.13. Animal Studies

All animal studies were approved by the Institutional Animal Care and Use Committee at the University of California, Davis (protocol # 20716; approved on September 6, 2018). For the efficacy study, C57BL/6J mice (4–6 weeks) were bilaterally, s.c injected with 0.3 × 10^6^ KPC cells/tumor suspended in 0.1 mL sterile PBS. When KPC cells reached palpable tumor size (~300 mm^3^), mice (n = 5/group) were divided randomly into four groups. Mice were either given vehicle, EGCG 10 mg/kg, 7x/week by intraperitoneal injection (I.P.) injections, gemcitabine 100 mg/kg, 2x/week by i.p injections, or EGCG in combination with gemcitabine at the above doses. The dose of EGCG was based on our previous studies [11,27]. Mice were treated for 16 days. Tumor size and body weight were measured every two days, and tumor size was determined by the equation length × width × (length + width/2) × 0.56, in millimeters [32]. At the end of the study, animals were euthanized by carbon dioxide asphyxiation, and tumor weights measured. Tumor and liver tissues were collected for analysis. For the drug combination toxicity study, C57BL/6J mice (n = 4/group) were treated either with PBS (vehicle control), or EGCG 10 mg/kg, 7x/week by i.p injections, in combination with gemcitabine 100 mg/kg, 2x/week by i.p injections. On day 16, mice were euthanized, blood was drawn, serum was collected, and a liver-kidney function panel was performed.

### 4.14. Immunohistochemistry

Immunohistochemical staining for for Ki-67 (Cat #12202) and cleaved Caspase-3 (Cat #9661, both from Cell Signaling Technology, Danvers, MA, USA) was performed as previously described [33]. Briefly, paraffin-embedded sections (5 μm thick) were deparaffinized and rehydrated, followed by antigen retrieval performed by microwave-heating in 0.01 M citrate buffer (pH 6.0). 3% H_2_O_2_ was used to block endogenous peroxidase activity for 10 min at room temperature. Slides were blocked for 60 min with serum, and incubated with primary antibody overnight at 4 °C. The following morning, slides were washed thrice with PBS, and then incubated with the biotinylated secondary antibody and the streptavidin-biotin complex (Invitrogen, Carlsbad, CA, USA) for 1 h of each at room temperature. After washing with PBS three times, slides were stained with 3,3′-Diaminobenzidine tetrahydrochloride hydrate (DAB) solution, and then counterstained with hematoxylin. Images were taken at 100× magnification. At least five fields per sample were scored and analyzed using Image J software (v1.46, NIH, Bethesda, MD, USA).

### 4.15. Statistical Analysis

Data were obtained from at least three independent biological experiments and results expressed as mean ± SD. One-way analysis of variance (ANOVA) and the Duncan test were used to analyze differences among multiple groups. *T*-tests were performed to compare the difference between two groups. *p* < 0.05 was regarded as being statistically significant.

## 5. Conclusions

EGCG strongly suppresses glycolysis through the inhibition of PFK, an effect that is ROS-dependent. In addition, EGCG presents a strong additive effect when combined with gemcitabine in pancreatic xenografts by further inhibiting glycolysis and affecting cell kinetics. Although additional studies to validate the above findings in complemmentary preclinical models of pancreatic cancer are warranted, our results suggest that EGCG is a useful combination partner of gemcitabine in pancreatic cancer treatment.

## Supplementary Materials

The following are available online at https://www.mdpi.com/2072-6694/11/10/1496/s1, Figure S1: EGCG reduced cell glycolysis, glycolytic capacity and glycolytic reserve in Panc-1, MIA PaCa-2 and KPC cells. Figure S2: Effect of EGCG on HK2 and LDHA protein expression. Table S1: Cell growth combination effects of EGCG with various chemotherapeutics in MIA PaCa-2 and Panc-1 cells. Table S2: Serum levels of multiple biochemical enzymes and markers of liver and kidney function for control and EGCG plus gemcitabine the end of the treatment period. Figure S3: Mice body weight progression for control and gemcitabine+EGCG treated groups. Figure S4. Western blot images with molecular weight for PFKP and PKM2 shown in Figure 2B. Figure S5. Western blot images with molecular weight for PFKP and PKM2 shown in Figure 2C. Figure S6. Western blot images with molecular weight for PFKP and PKM2 shown in Figure 2D and Figure 2E. Figure S7. Western blot images with molecular weight for PFKP and PKM2 shown in Figure 3D. Figure S8. Western blot images with molecular weight for p-Chk1, p53, p21, cdc2 and CyclinB1 shown in Figure 6B. Figure S9. Western blot images with molecular weight for Caspase 3, Caspase 7, Caspase 9 and PARP shown in Figure 6D. Figure S10. Western blot images with molecular weight for Bcl-xl, Bad, XIAP, Survivin shown in Figure 6E. Figure S11. Western blot images with molecular weight for PFKP and PKM2 shown in Figure 7B. Figure S12. Western blot images with molecular weight for HK2 and LDHA shown in Figure S2.
