# Supplementary material for: Targeting Glycolysis with Epigallocatechin-3-Gallate Enhances the Efficacy of Chemotherapeutics in Pancreatic Cancer Cells and Xenografts
Source: Cancers (Basel). 2019 Oct 5;11(10):1496. doi: 10.3390/cancers11101496 (PMC6826788; doi:10.3390/cancers11101496)
Supplement: Supplementary file 1 [file cancers-11-01496-s001.pdf]

Supplementary Materials: Targeting Glycolysis with Epigallocatechin-3-Gallate (EGCG) Enhances the Efficacy of Chemotherapeutics in Pancreatic Cancer Cells and Xenografts

Ran Wei, Robert M. Hackman, Yuefei Wang and Gerardo G. Mackenzie

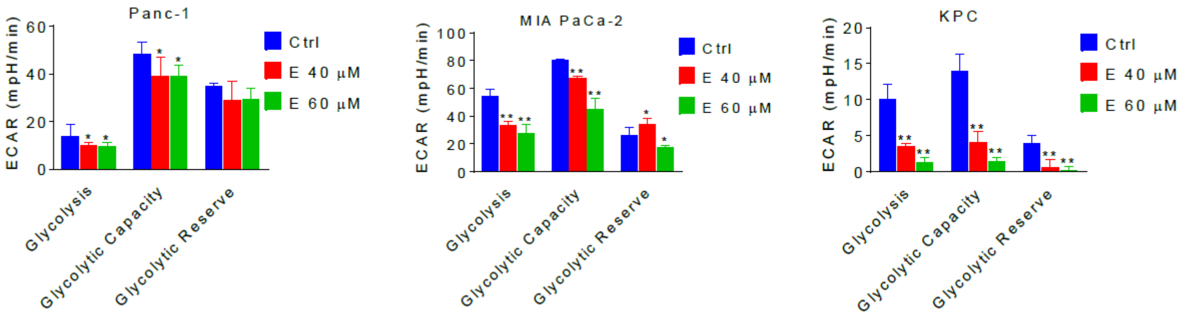

Figure S1. EGCG reduced cell glycolysis, glycolytic capacity and glycolytic reserve in Panc-1, MIA PaCa-2 and KPC cells. Results are presented as the mean ± SD of ECAR. * p <0.05, ** p < 0.01 vs. control.

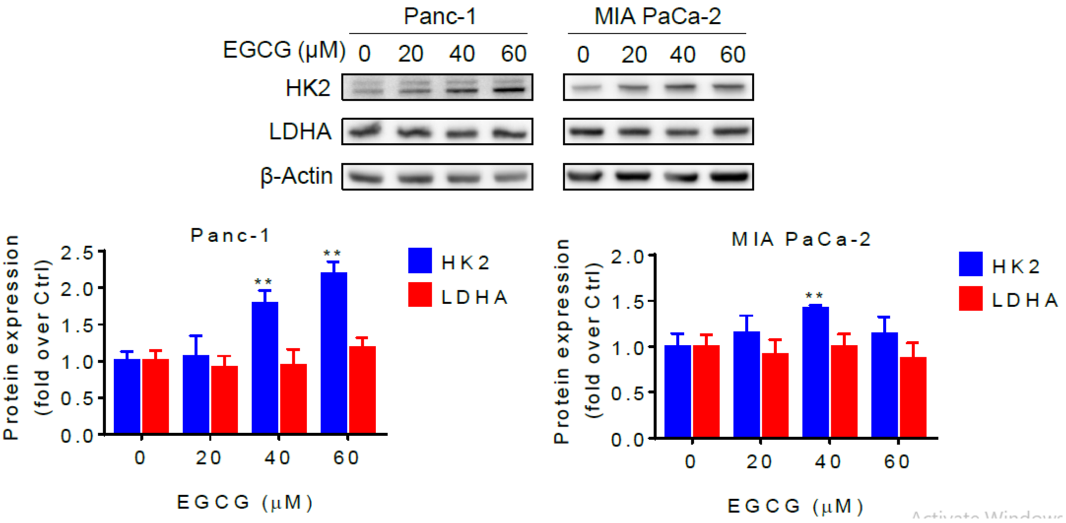

Figure S2. Effect of EGCG on HK2 and LDHA protein expression. Results are expressed as fold over control. * $p < 0.05$, ** $p < 0.01$ vs. control.

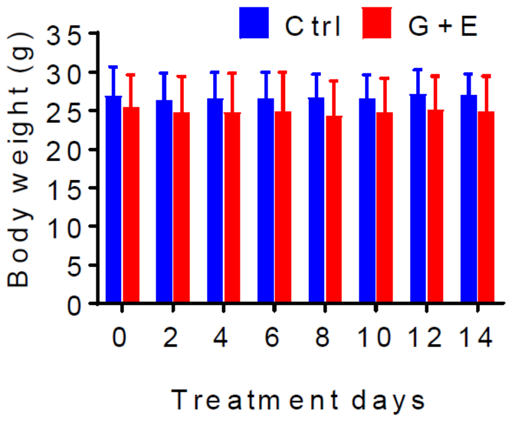

Figure S3. Mice body weight progression for control and EGCG plus gemcitabine treated groups. Results are presented as the mean ± SD.

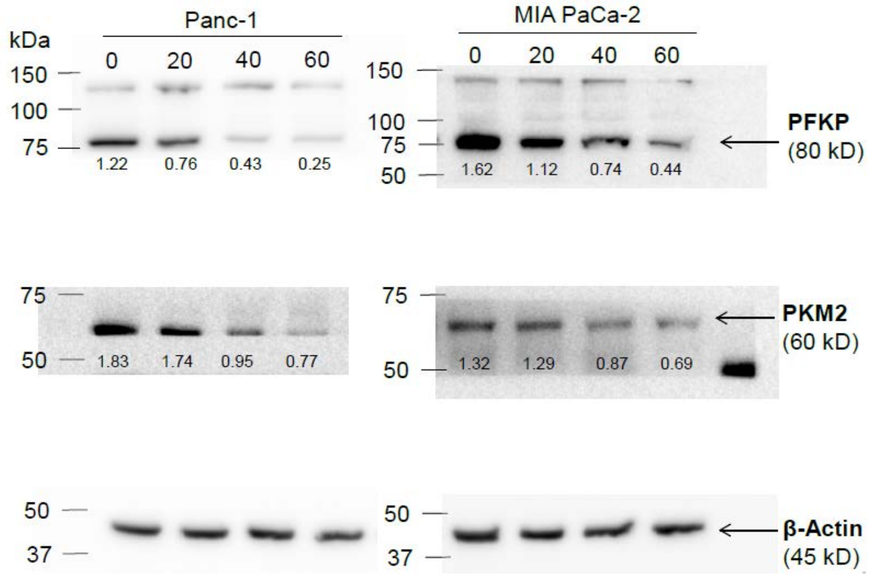

Figure S4. Western blot images with molecular weight for PFKP and PKM2 shown in Figure 2B.

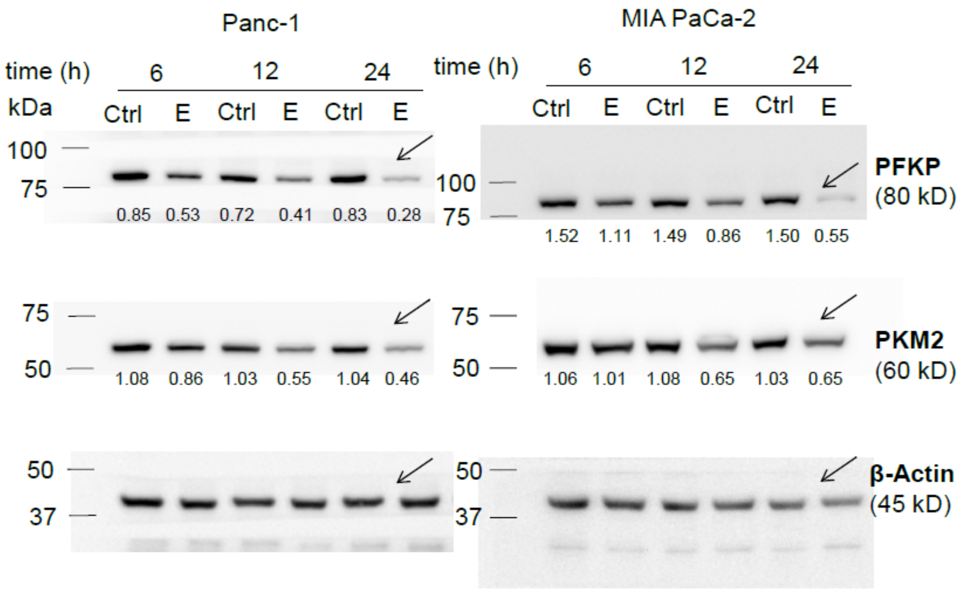

Figure S5. Western blot images with molecular weight for PFKP and PKM2 shown in Figure 2C.

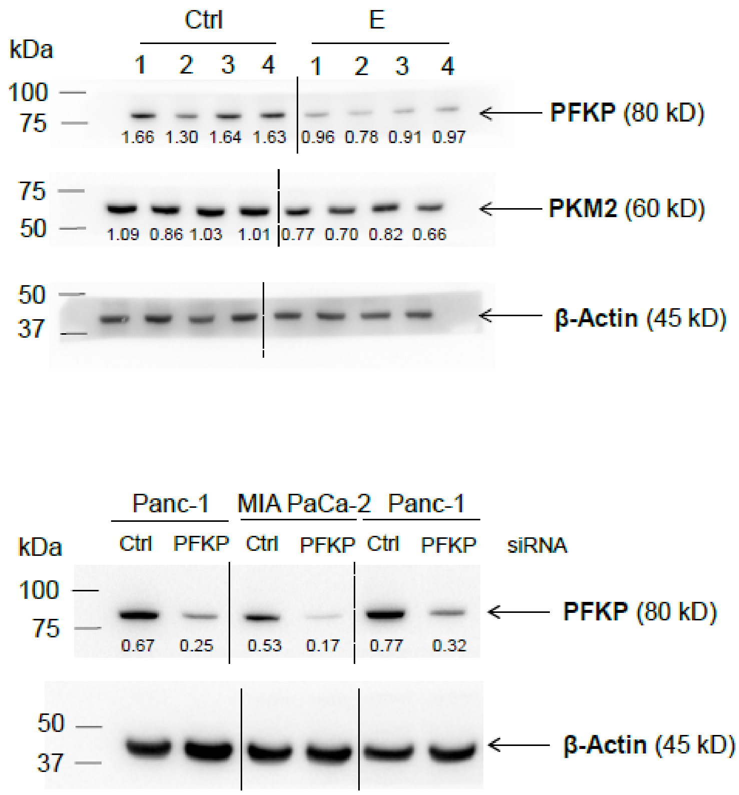

Figure S6. Western blot images with molecular weight for PFKP and PKM2 shown in Figure 2D and Figure 2E.

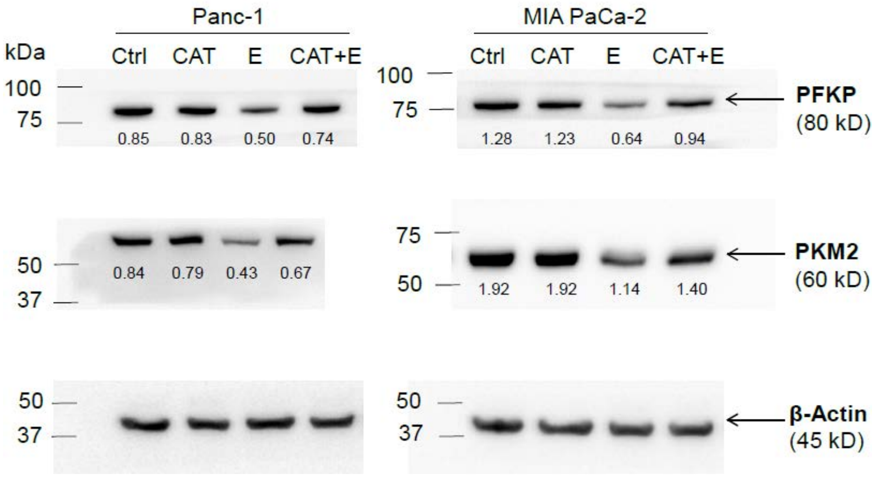

Figure S7. Western blot images with molecular weight for PFKP and PKM2 shown in Figure 3D.

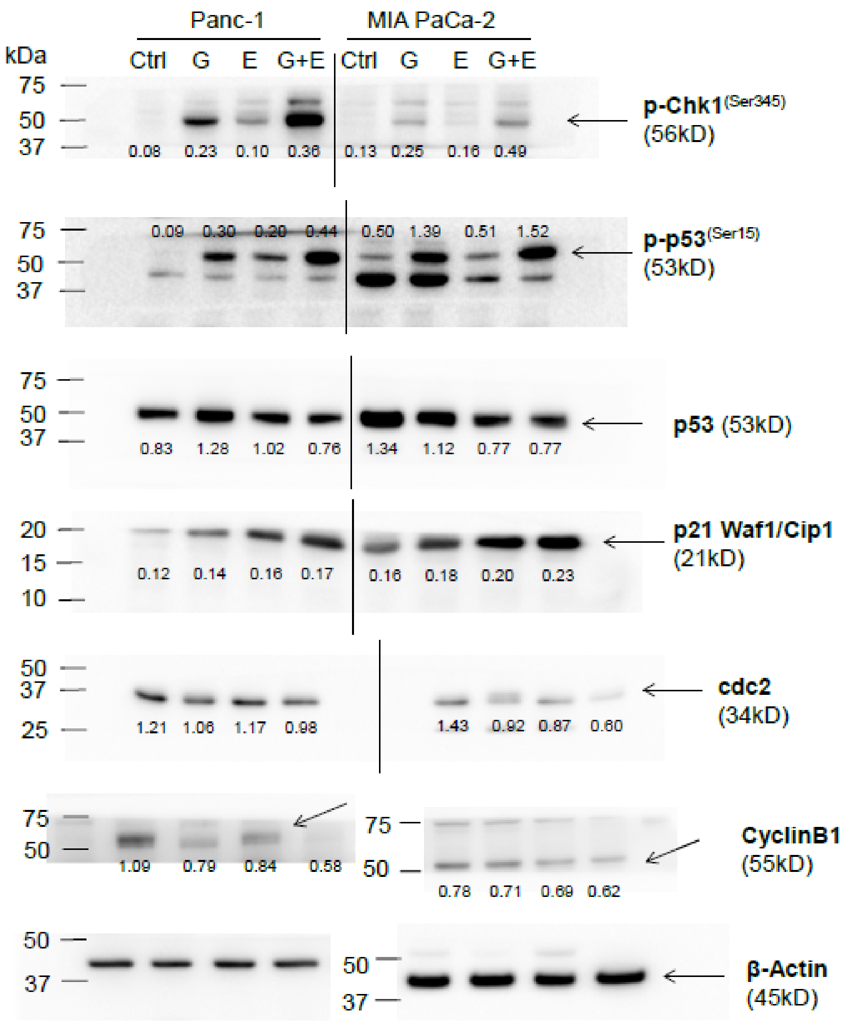

Figure S8. Western blot images with molecular weight for p-Chk1, p53, p21, cdc2 and CyclinB1 shown in Figure 6B.

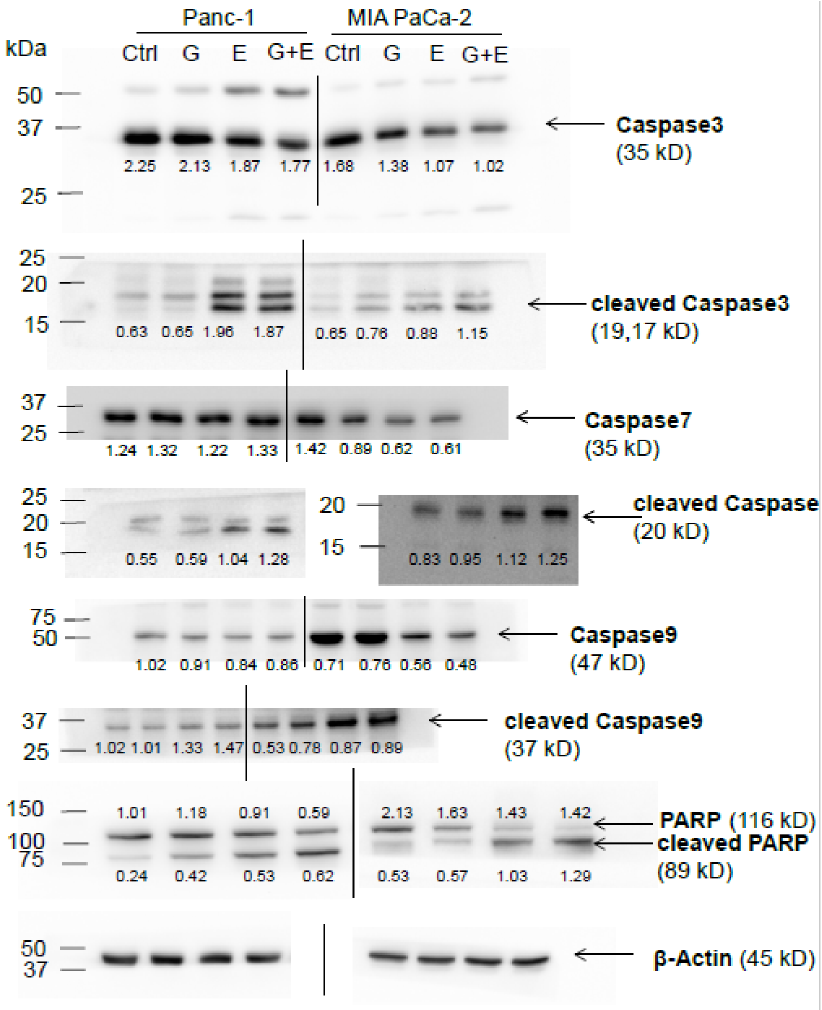

Figure S9. Western blot images with molecular weight for Caspase3, Caspase7, Caspase9 and PARP shown in Figure 6D.

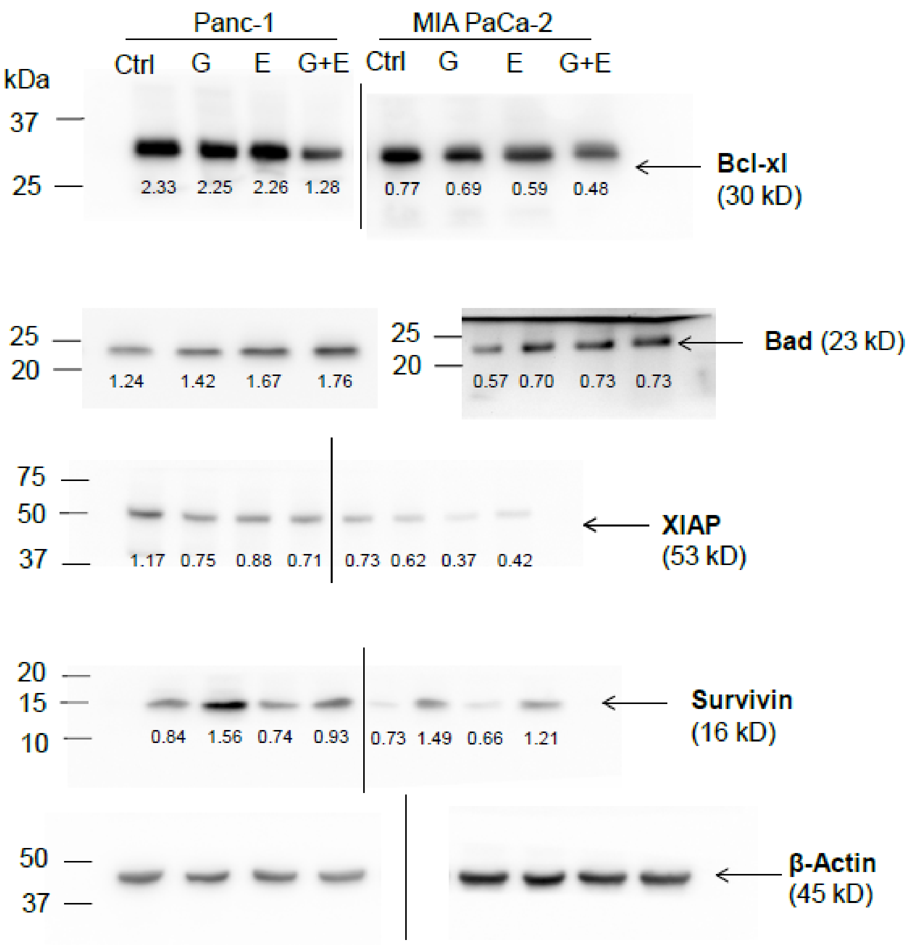

Figure S10. Western blot images with molecular weight for Bcl-xl, Bad, XIAP, Survivin shown in Figure 6E.

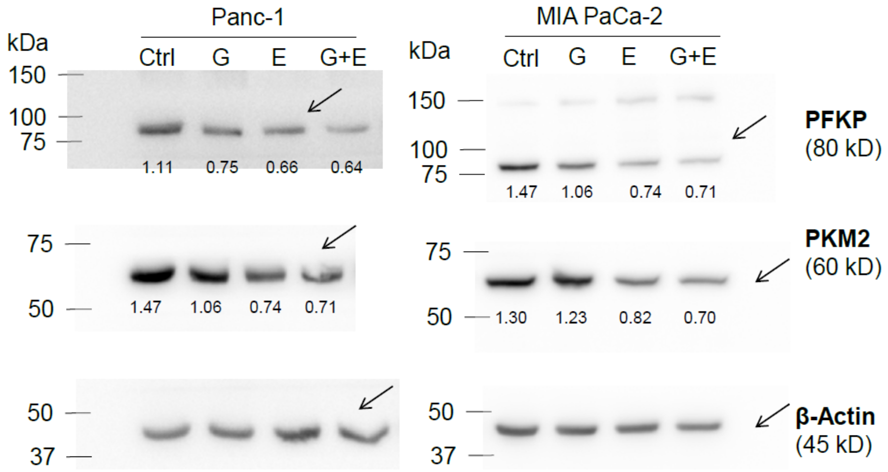

Figure S11. Western blot images with molecular weight for PFKP and PKM2 shown in Figure 7B.

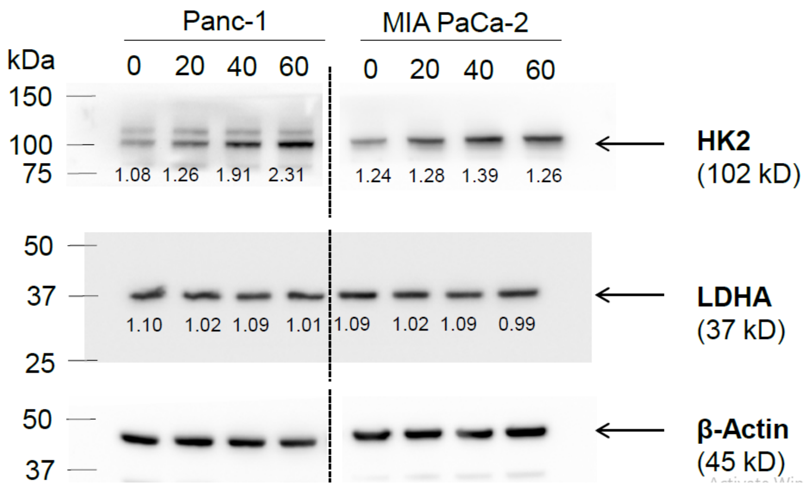

Figure S12. Western blot images with molecular weight for HK2 and LDHA shown in Figure S2.

Table S1. Cell growth combination effects of EGCG with various chemotherapeutics.

		**EGCG (μM)**					
**Panc-1**		20.00		40.00		60.00	
	IR	IR	CI	IR	CI	IR	CI
Abraxane (nM)	—	37.01 ± 3.33	—	52.88 ± 5.87	—	57.89 ± 3.28	—
1	19.53 ± 2.52	32.62 ± 1.99	1.55	54.19 ± 5.23	0.89	59.16 ± 4.09	1.02
10	37.02 ± 3.77	51.85 ± 1.50	0.93	62.72 ± 4.91	0.73	65.15 ± 4.92	0.87
20	56.55 ± 2.83	62.25 ± 7.21	0.66	65.78 ± 2.84	0.74	68.52 ± 6.53	0.81
40	57.70 ± 4.26	65.08 ± 4.44	0.84	64.18 ± 4.67	1.15	68.51 ± 3.65	1.03
		EGCG (μM)					
Panc-1		20.00		40.00		60.00	
	IR	IR	CI	IR	CI	IR	CI
5-Fluorouracil (μM)	-	31.54 ± 2.71	-	52.86 ± 2.06	-	59.41 ± 3.34	-
1	24.96 ± 4.22	27.13 ± 3.97	2.29	65.08 ± 7.81	0.58	73.73 ± 3.71	0.58
10	54.45 ± 2.10	57.80 ± 4.21	0.62	72.60 ± 6.21	0.45	74.08 ± 3.41	0.60
20	50.33 ± 4.73	59.34 ± 7.13	0.76	73.50 ± 4.09	0.46	74.25 ± 5.64	0.62
40	53.26 ± 5.28	58.27 ± 6.54	1.30	72.61 ± 3.83	0.56	73.21 ± 2.70	0.73
		EGCG (μM)					
Panc-1		20.00		40.00		60.00	
	IR	IR	CI	IR	CI	IR	CI
Gemcitabine (nM)	-	34.01 ± 5.34	-	51.67 ± 6.96	-	56.01 ± 5.89	-
1	8.19 ± 0.20	39.29 ± 4.88	0.82	53.06 ± 7.89	0.84	57.01 ± 7.09	1.04
10	27.62 ± 4.64	63.78 ± 3.11	0.28	68.15 ± 6.17	0.42	68.17 ± 4.87	0.61
20	27.12 ± 5.37	71.26 ± 6.92	0.20	67.16 ± 6.13	0.47	65.52 ± 4.37	0.74
40	39.52 ± 7.99	70.87 ± 6.57	0.25	69.53 ± 3.54	0.45	66.26 ± 5.52	0.77
		EGCG (μM)					
Panc-1		20.00		40.00		60.00	
	IR	IR	CI	IR	CI	IR	CI
Irinotecan (μM)	-	38.12 ± 3.55	-	52.04 ± 2.21	-	56.33 ± 0.80	-
1	16.51 ± 2.50	50.97 ± 7.44	0.55	52.92 ± 5.16	0.92	54.60 ± 4.75	1.23
10	35.57 ± 3.91	56.38 ± 7.14	0.82	52.18 ± 6.59	1.51	54.02 ± 2.58	1.76
20	54.09 ± 5.76	54.24 ± 8.88	1.47	51.79 ± 3.43	2.16	58.26 ± 2.51	1.78
40	71.67 ± 4.08	64.44 ± 7.86	1.34	65.52 ± 3.85	1.45	93.83 ± 0.87	0.07
Panc-1		EGCG (μM)					

		20.00		40.00		60.00	
	IR	IR	CI	IR	CI	IR	CI
Oxaliplatin (μM)	-	31.45 ± 7.85	-	55.15 ± 5.41	-	60.02 ± 3.90	-
1	13.52 ± 5.75	59.85 ± 3.62	0.38	61.28 ± 3.71	0.71	63.44 ± 2.83	0.97
10	42.60 ± 6.54	62.87 ± 4.88	0.45	62.18 ± 3.03	0.80	64.80 ± 6.06	1.02
20	43.77 ± 1.97	63.01 ± 6.43	0.57	60.30 ± 5.36	1.03	66.07 ± 6.61	1.06
40	51.21 ± 7.82	57.08 ± 3.90	1.20	65.87 ± 4.77	0.97	64.81 ± 2.97	1.34

		EGCG (μM)					
MIA PaCa-2		20.00		40.00		60.00	
	IR	IR	CI	IR	CI	IR	CI
Abraxane (nM)	-	31.51 ± 2.19	-	51.83 ± 1.90	-	63.01 ± 3.23	-
1	6.86 ± 1.23	30.68 ± 1.74	1.24	63.07 ± 2.74	0.73	62.78 ± 4.01	1.08
10	48.06 ± 3.78	55.76 ± 8.00	1.24	60.99 ± 6.01	1.39	65.79 ± 3.88	1.47
20	68.52 ± 6.94	63.50 ± 7.55	1.55	65.74 ± 7.26	1.72	70.18 ± 4.81	1.70
40	83.25 ± 4.75	74.90 ± 4.33	1.72	69.25 ± 8.33	2.47	74.25 ± 5.48	2.21

		EGCG (μM)					
MIA PaCa-2		20.00		40.00		60.00	
	IR	IR	CI	IR	CI	IR	CI
5-Fluorouracil (μM)	-	36.48 ± 4.95	-	54.01 ± 3.03	-	62.33 ± 6.60	-
1	16.04 ± 4.70	34.67 ± 7.11	1.25	54.79 ± 4.72	0.97	64.93 ± 8.74	0.92
10	44.24 ± 6.82	42.15 ± 3.40	1.64	59.59 ± 7.65	0.99	66.77 ± 7.45	0.96
20	46.65 ± 8.23	46.87 ± 5.31	1.82	60.94 ± 6.50	1.12	67.55 ± 8.78	1.03
40	57.13 ± 4.22	58.64 ± 6.70	1.34	66.46 ± 4.05	1.06	67.54 ± 4.14	1.26

		EGCG (μM)					
MIA PaCa-2		20.00		40.00		60.00	
	IR	IR	CI	IR	CI	IR	CI
Gemcitabine (nM)	-	38.71 ± 4.16	-	49.61 ± 3.22	-	60.57 ± 6.69	-
1	9.77 ± 2.48	37.69 ± 4.09	1.07	58.47 ± 2.48	0.71	60.66 ± 2.70	0.94
10	32.73 ± 7.73	53.31 ± 3.29	0.60	56.67 ± 5.56	0.88	77.53 ± 8.33	0.36
20	37.54 ± 4.29	56.82 ± 4.92	0.60	77.76 ± 4.13	0.26	78.91 ± 5.01	0.34
40	42.03 ± 6.03	51.28 ± 3.52	1.16	73.27 ± 5.62	0.41	75.01 ± 7.71	0.50

		EGCG (μM)					
MIA PaCa-2		20.00		40.00		60.00	
	IR	IR	CI	IR	CI	IR	CI
Irinotecan (μM)	-	36.70 ± 2.54	-	55.14 ± 3.80	-	66.06 ± 3.46	-
1	31.98 ± 7.35	35.12 ± 4.12	1.77	63.76 ± 5.17	0.93	72.11 ± 6.10	0.90
10	68.95 ± 5.87	58.14 ± 6.49	3.07	66.53 ± 8.14	2.44	69.42 ± 5.30	2.42
20	85.75 ± 6.63	59.53 ± 7.09	5.35	67.09 ± 6.43	4.12	72.55 ± 7.25	3.40
40	94.80 ± 1.92	85.26 ± 2.47	2.44	79.43 ± 3.02	3.90	81.12 ± 3.48	3.66

		EGCG (μM)					
MIA PaCa-2		20.00		40.00		60.00	
	IR	IR	CI	IR	CI	IR	CI
Oxaliplatin (μM)	-	34.61 ± 3.26	-	60.10 ± 6.27	-	67.31 ± 3.61	-
1	32.91 ± 9.41	34.82 ± 6.24	1.86	70.19 ± 6.85	0.69	76.80 ± 3.74	0.76
10	62.83 ± 4.02	64.34 ± 5.27	1.22	68.31 ± 6.88	1.28	76.86 ± 3.29	0.99
20	67.41 ± 6.32	63.62 ± 4.01	2.17	67.42 ± 7.85	1.99	74.38 ± 3.91	1.49
40	78.77 ± 4.91	64.28 ± 2.13	3.73	70.23 ± 3.59	2.64	78.10 ± 3.74	1.60

The combination effect was evaluated by the combination index (CI) obtained from the Chou-Talalay method. EGCG increased the sensitivity of gemcitabine by further increasing the inhibition rate (IR)

in both Panc-1 and MIA PaCa 2 cells. Cells were treated by EGCG together with different drugs for 72 h, and then cell growth was evaluated. Results are presented as the mean ± SD.

Table S2. Serum levels of multiple biochemical enzymes and markers of liver and kidney function for control and EGCG plus gemcitabine the end of the treatment period.

	**Alanine**	**Alkaline**	**Aspartate**	**Blood**	**Creatinine**	**Total**	**Total**
	Transaminase	Phosphatase	Transaminase	Urea Nitrogen		Bilirubin	Protein
	U/L	U/L	U/L	mg/dL	mg/dL	mg/dL	g/dL
Reference Range	0 – 403	49 – 172	0 – 552	15.2 – 34.7	0.0 – 0.3	0.0 – 0.2	4.7 – 6.1
Ctrl	29.93 ± 2.53	117.08 ± 43.57	52.83 ± 5.36	29.38 ± 1.34	0.12 ± 0.02	0.05 ± 0.03	5.70 ± 0.36
G + E	58.23 ± 25.94	147.35 ± 38.03	106.25 ± 35.40	33.20 ± 5.16	0.14 ± 0.02	0.04 ± 0.02	5.83 ± 0.29
